# Morphological alterations of T24 cells on flat and nanotubular TiO_2_ surfaces

**DOI:** 10.3325/cmj.2012.53.577

**Published:** 2012-12

**Authors:** Roghayeh Imani, Doron Kabaso, Mateja Erdani Kreft, Ekaterina Gongadze, Samo Penič, Kristina Eleršič, Andrej Kos, Peter Veranič, Robert Zorec, Aleš Iglič

**Affiliations:** 1 Faculty of Electrical Engineering, University of Ljubljana, Ljubljana, Slovenia; 2 Institute of Cell Biology, Faculty of Medicine, University of Ljubljana, Ljubljana, Slovenia; 3 Laboratory of Neuroendocrinology-Molecular Cell Physiology, Faculty of Medicine, University of Ljubljana, Ljubljana, Slovenia; 4 Celica Biomedical Center, Ljubljana, Slovenia; 5 J. Stefan Institute, Ljubljana, Slovenia; * The first two authors contributed equally.

## Abstract

**Aim:**

To investigate morphological alterations of malignant cancer cells (T24) of urothelial origin seeded on flat titanium (Ti) and nanotubular titanium dioxide (TiO_2_) nanostructures.

**Methods:**

Using anodization method, TiO_2_ surfaces composed of vertically aligned nanotubes of 50-100 nm diameters were produced. The flat Ti surface was used as a reference. The alteration in the morphology of cancer cells was evaluated using scanning electron microscopy (SEM). A computational model, based on the theory of membrane elasticity, was constructed to shed light on the biophysical mechanisms responsible for the observed changes in the contact area of adhesion.

**Results:**

Large diameter TiO_2_ nanotubes exhibited a significantly smaller contact area of adhesion (*P* < 0.0001) and had more membrane protrusions (eg, microvilli and intercellular membrane nanotubes) than on flat Ti surface. Numerical membrane dynamics simulations revealed that the low adhesion energy per unit area would hinder the cell spreading on the large diameter TiO_2_ nanotubular surface, thus explaining the small contact area.

**Conclusion:**

The reduction in the cell contact area in the case of large diameter TiO_2_ nanotube surface, which does not enable formation of the large enough number of the focal adhesion points, prevents spreading of urothelial cells.

The material and topographical characteristics of the contact surface affect the functional activity of cells (1-7). Titanium is the most widely used material in numerous medical applications, because it is non-toxic. The titanium surface has recently been modified by a self-assembled layer of vertically oriented titanium oxide (TiO_2_) nanotubes with diameters between 15 nm and 100 nm (8-10). It was revealed that cell adhesion, spreading, growth, and differentiation was maximally induced on 15 nm nanotubes, but hindered on 100 nm nanotubes, which facilitated cell death (8,9). These results suggest that magnitude of TiO_2_ nanotube diameter has an important role in cell adhesion and cell growth, and that the mechanics of the formation of focal adhesions is similar between different types of cells. The aim of the present study is to analyze effects of flat titanium and nanotubular TiO_2_ surfaces on the morphology of malignant cancer cells (T24) of urothelial origin.

Using anodization method, vertically aligned TiO_2_ nanotubular surfaces were produced (Figure 1). The produced nanotubes had a large diameter (50-100 nm). The T24 cells grown on the nanotubular surface had a smaller top view diameter and more membrane protrusions than the counterpart on the flat titanium surface. A computational model was constructed to shed light on the biophysical mechanism underlying the observed morphologic changes. The underlying hypothesis is that the low density of TiO_2_ nanotube edges could not facilitate the cell adhesion, ie, the formation of large enough number of focal adhesion points. Mathematically, the density of negative charges is predicted to be greatest at sharp edges (7), which would then facilitate the mediated electrostatic interactions between the TiO_2_ nanotube surface and membrane proteins at the focal contact. Using numerical simulations of membrane dynamics, it is revealed that low adhesion of the membrane to the large diameter nanotubular surface is not sufficient to counterbalance the loss of entropic energy during the clustering of proteins at a focal contact, and consequently, the increased membrane bending energy does not favor intensive spread of cancer cells on the large diameter TiO_2_ nanotube surface underneath.

**Figure 1:**
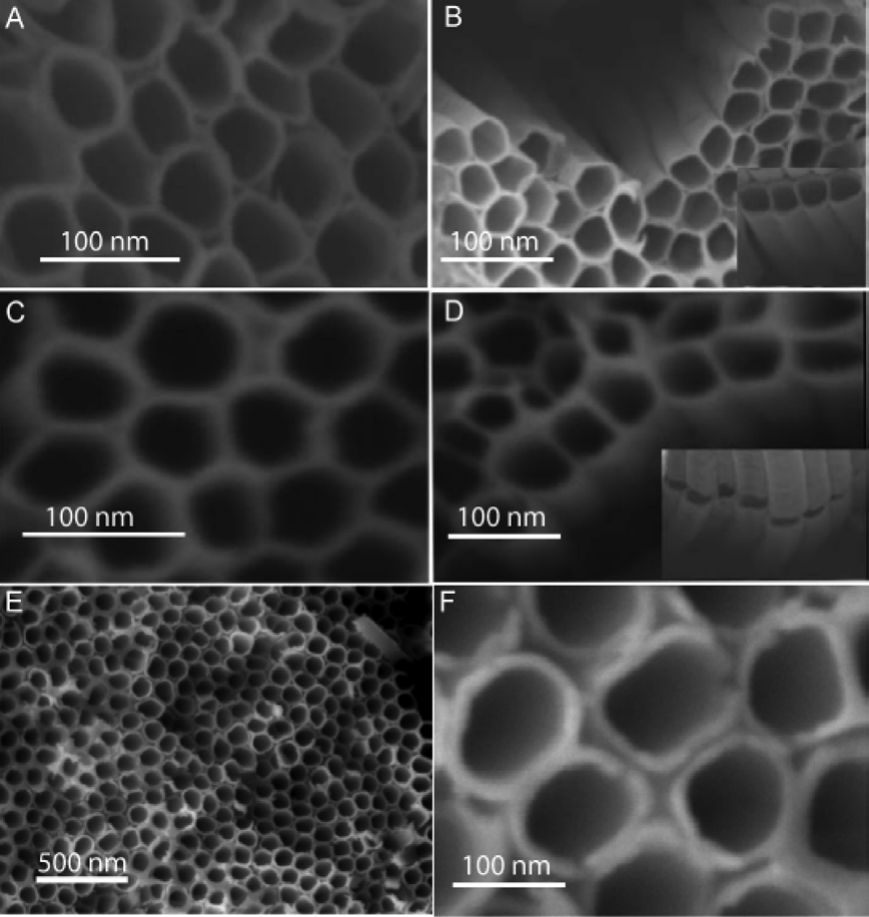
Scanning electron microscopy images of surface layers of self-assembled vertically aligned TiO_2_ nanotubes synthesized by anodization method. Ethylene glycol solution with 0.3 wt % NH_4_F and 1% volume water was used in preparation. High-resolution scanning electron microscopy FEG-SEM 7600F from JEOL was used. Images were taken at 100000× magnitude using low accelerating voltage of 2kV. The internal TiO_2_ tube diameter was around 50-60 nm (**A-D**) and 100 nm (**E-F**) while the length could be up to 10 micrometers (**B**).

## Material and methods

### TiO_2 _nanotube synthesis

TiO_2_ nanotubes were synthesized by anodization of Ti foil (thickness 0.25 mm; purity 99.5%; Sigma-Aldrich, St. Luis, MO, USA) in a two electrode electrochemical cell. Prior to nanotube synthesis, titanium foils were degreased by sonicating in acetone, methanol, and ethanol, then rinsed with deionized water and dried in a nitrogen stream. The electrolyte was ethylene glycol containing 0.2 vol% H_2_O and 0.3 w% NH_4_F. Anodization was done at room temperature with titanium foil as anode and platinum foil as cathode. A DC power supply was used as the voltage source. The Ti foil was biased at 60 V and 80 V for 1 hour to grow a nanotubular TiO_2_ layer. After the electrochemical treatment, the samples were rinsed with deionized water and dried in air. A field-emission SEM was used for morphological characterization of samples (Figure 1).

### Urothelial cell culture

Human urothelial T24 cell line was cultured in a 1:1 mixture of Advanced-Dulbecco’s modified essential medium (Invitrogen, Gibco, Paisley, UK) and Ham’s F-12 medium (Sigma-Aldrich Corp.), supplemented with 10% fetal bovine serum (FBS, Gibco, Invitrogen, Carlsbad, CA, USA), 5 µg/ml insulin-transferrin-selenium supplement (Gibco), 100 µg/ml streptomycine, and 100 units/mL penicillin. Just at confluence, the cells were incubated in TrypLE Select (Gibco) at 37°C for 5 minutes, resuspended in the medium, centrifuged at 200 g for 5 minutes, and plated at seeding density of 5 × 10^4^ cells/cm^2^ on TiO_2_ nanotube and flat surfaces. The urothelial-TiO_2_ constructs were maintained for a week at 37°C in a humidified atmosphere of 5% CO_2_ (v/v) in air. After a week, the ultra-structural status of the urothelial cells growing on the TiO_2_ nanotube and flat surfaces was analyzed.

### Scanning electron microscopy

A scanning electron microscope was employed for morphological characterization of urothelial cells adhered to TiO_2_ nanotubular and flat titanium surfaces. Urothelial-titanium/TiO_2_ constructs were prepared for scanning electron microscopy (SEM) as described previously (11). In brief, after a week of culturing, the urothelial-titanium/TiO_2_ constructs were fixed in 4% (w/v) paraformaldehyde and 2.5% (v/v) glutaraldehyde in 0.1 M cacodylate buffer, pH 7.4 for 2 hours 45 minutes. The fixation was followed by overnight rinsing in 0.1 M cacodylate buffer. The samples were then postfixed in 1% (w/v) osmium tetroxide for 1 hour at 4°C. After dehydration through a graded series of acetone, the samples were dried at the critical point, gold sputtered, and observed in a Jeol 840A (Jeol, Tokyo, Japan) scanning electron microscope.

### Statistical analysis

**Figure 2:**
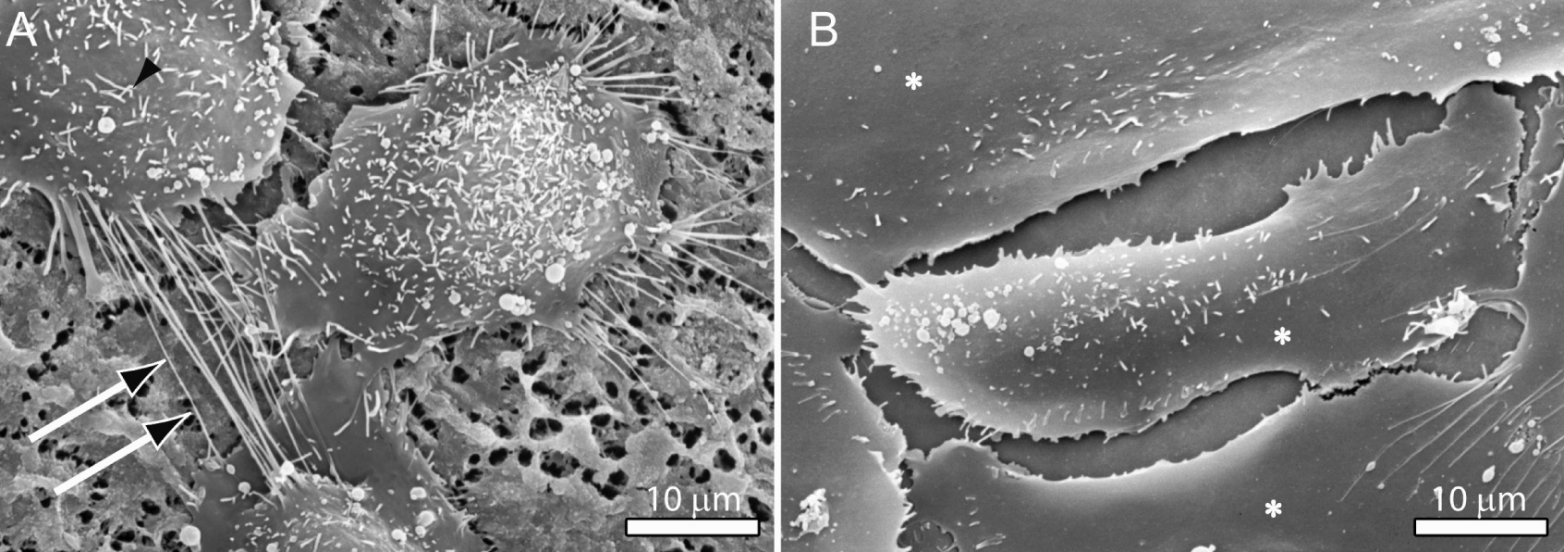
Urothelial T24 cells on (**A**) large diameter (of 50-100 nm diameter) TiO_2_ nanotubes and on (**B**) flat titanium surfaces. The surface topography of urothelial cells reveals smaller cell diameter and numerous membrane protrusions (eg, nanotubular structures – thin arrows and pleomorphic microvilli – arrowheads) on the large diameter TiO_2_ nanotubes (**A**), whereas, on the flat titanium surface, the cells are considerably larger in size and the cell membrane is smoother (asterisks) (**B**). Scale bars: 10 μm.

The surface area of T24 cells was measured using surface area tool in ImageJ. A total of eleven SEM micrographs were analyzed for each of the cell groups in a single micrograph. The two images in Figure 2 have a resolution of 1200 dpi (472.441 pixels/cm), while the rest of the images have a resolution 600 dpi (236.22 pixels/cm). The figures were 6.8 cm in width and 4.87 cm in height. The statistical analysis between T24 cells grown on the nanotubular surface and T24 cells grown on the flat TiO_2_ surface was done using a *t* test for independent samples. Statistical significance was set at the *P* < 0.0001. Data are reported as means ± standard deviation.

### The model

**Figure 3:**
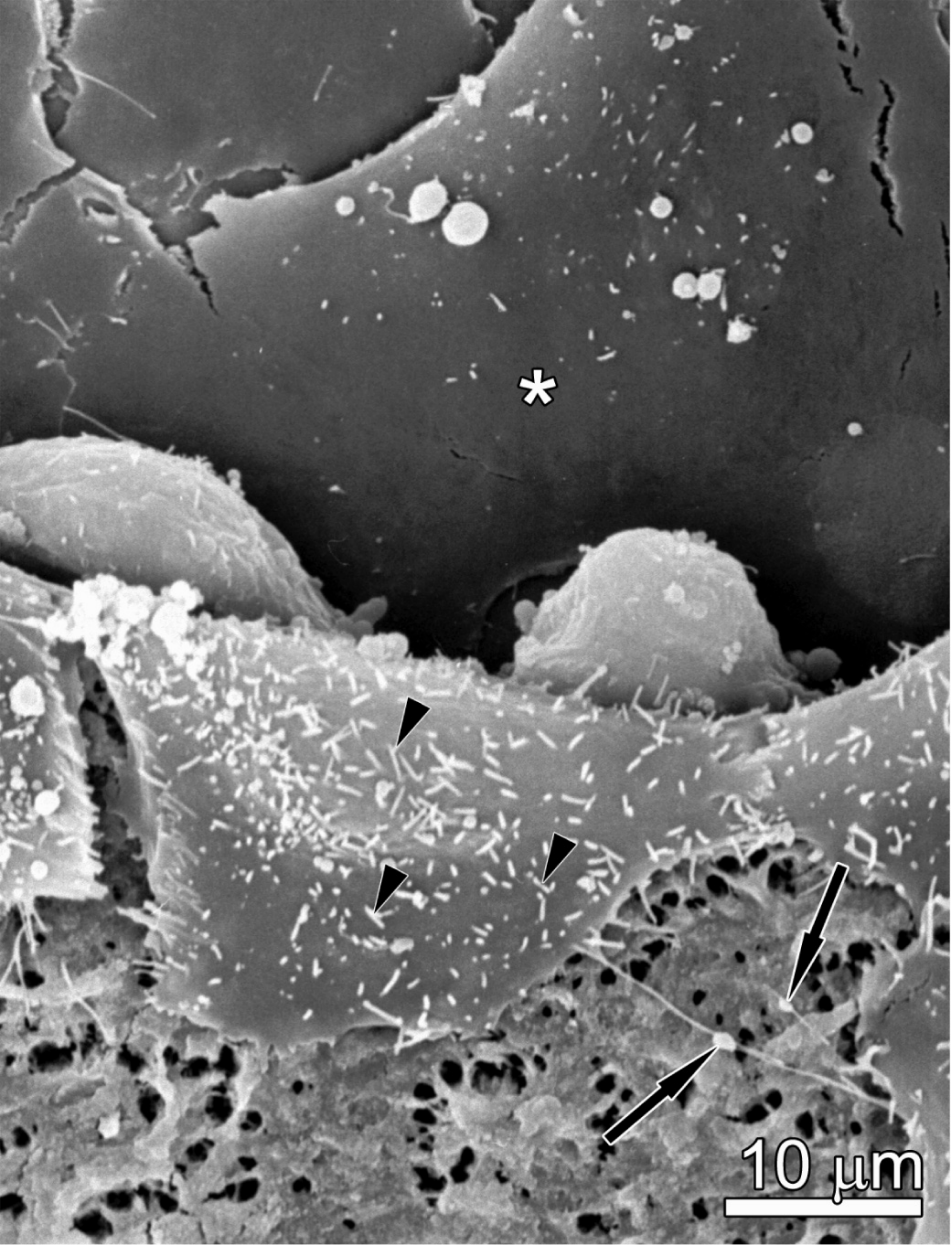
Urothelial T24 cells on TiO_2_ and Ti scaffold. One half of the scaffold was flat titanium and another half was constructed into TiO_2_ nanotubes. Urothelial cells growing on the TiO_2_ nanotube surface are smaller, more spherical (ie, less flattened) and have numerous long membrane exvaginations. Their apical surface is covered by pleomorphic microvilli (arrowheads). The gondola-like structures are indicated by arrows. The urothelial cells on the flat titanium surface are flatter than cells on the large diameter TiO_2_ nanotube surface. Their apical surface is smooth (asterisk), only few microvilli are seen. Scale bar: 10 μm.

To investigate the dynamics of membrane growth on nanotubular TiO_2_ scaffolds and flat titanium surface, we constructed a simple model based on the membrane free energy. The idea behind the model is that the membrane attains the shape that corresponds to the minimum free energy. This quasi-equilibrium shape is derived by the minimization of the membrane free energy. The equation of motion (Equation 1) describes the interplay of the adhesion, bending, and cross-interaction energies. The modeled membrane constituent is an integrin molecule that facilitates (mediates) membrane adhesion, and due to the large extracellular part, it has a positive spontaneous curvature. In the model, the overall number of integrins was conserved, moving laterally over the cell membrane. The theoretical model is an extension of our previous theoretical models (7,10,12-14). The investigated shape was a segment of the cell (membrane) outer contour, which was initially flat (Figure 3). The membrane shape was under the constraint of translational symmetry. Moreover, we assumed that the membrane curvature along the direction perpendicular to the contour was roughly constant, and thus, it entered our calculation as a modified membrane tension. The types of surfaces were: 1) a flat titanium surface; 2) a small diameter (15 nm) TiO_2_ nanotubular surface; and 3) a large diameter (100 nm) nanotubular surface. The TiO_2_ nanotubular surfaces were modeled explicitly by including a vertical intersection of the nanotubes.

### Equations of membrane dynamics

Our model investigated the observed differences in the cell diameter between two different types of TiO_2_ nanotube surfaces. The membrane free energy expression was employed to derive the equations of motion of a cell membrane contour and the density distribution of integrins. The membrane free energy in our dynamical model was:


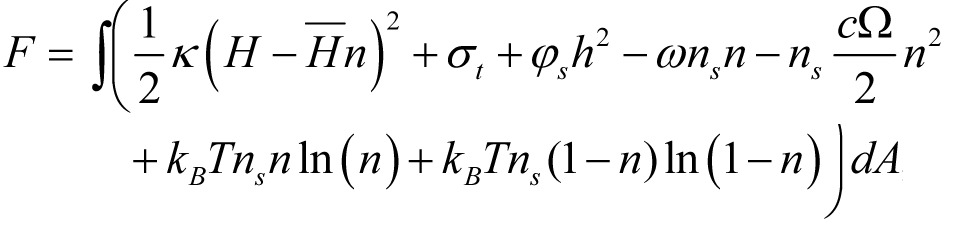
 (1)

where the first term gives the bending energy due to the mismatch between the membrane curvature and the membrane spontaneous curvature of integrins, κ is the bending modulus, *H* is the local membrane mean curvature, *Ħ* is the intrinsic mean curvature due to embedded integrin, and *n* is the area fraction density of integrins (relative density) (7). The second term σ*_t_* is Lagrange multiplier (having the units of membrane surface tension) for the conservation constraint of the total membrane area. The third term gives the energy due to force of the cytoskeleton inside cells and φ_s_ is the restoring cytoskeleton spring constant. The fourth term is the negative binding potential of integrins (ω>0). The fifth term describes direct interaction Bragg-Williams term between neighboring integrins. The sixth and seventh terms are the entropic energies, taking into account the finite areas of integrins. Ω is the direct-interaction Bragg-Williams constant (Ω>0), *c* is the number of the nearest neighbor integrins, *h = h(s)* describes the magnitudes of small deformations from the flat membrane, *n_s_ = 1/a_0_* is the saturation area density of integrins, where *a_0_* is the cross-section area of a single integrin, k_B_T is thermal energy, and dA is the area element.

Next, we derived the equations of motion of the membrane contour using the differentiation of the free energy (Equation 1) with respect to the membrane amplitude (*h(s)*) and integrin concentration (*n*). To take into account the drag due to viscous forces, we assumed, for simplicity, only local friction forces with coefficient *ξ*. For the nearly flat membrane segment, the equation of motion of the membrane was given by


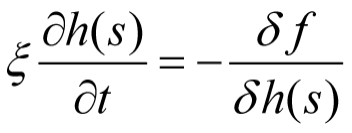
 (2)

where *t* is time and *f* is *F/dA*. Note that the force density


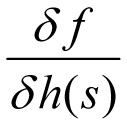


equals the membrane shape velocity times friction coefficient, but opposite in direction. Since the relative change in the *y* (ie, vertical, *y = h(s)*) direction is considerably greater than the change along the *x* (ie, horizontal) direction, we only consider the changes along the vertical *y* direction. We now calculate the dynamics of the integrin density, using the following conservation equation:


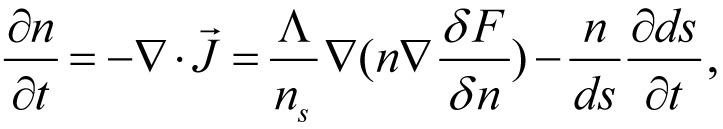
(3)

where Λ is the mobility of filaments, *Λ = D/ k_B_T*, *D* is the diffusion coefficient, and *J* is the total current of integrins on the membrane. For details of forces and fluxes derived from the differentiation of the free energy see our previous studies (12-14).

The results in the present study were calculated using numerical simulations of model system dynamics beyond linear limit. For simplicity, the boundary conditions on the nearly flat membrane were considered to be periodic. The following is the list of parameter values incorporated in our numerical simulations: φ_s_ = 0.008 gμm^-2^ s^-2^, κ = 100 k_B_T,  *Ħ*= 10 μm^-1^, *σ_t_* = 0.001 g s^-2^, ω = 1*10^−3^ gμm^2^s^-2^, *c* = 4, Ω = 3.5*10^−6^ gμm^2^s^-2^, *n_s_* = 100μm^-2^, ξ = 125s^-1^g, D = 0.005 μm^2^s^-1^. The binding coefficient ω was nonzero only above the nanotube edges, which was over segments of 20 nm in the case of the large diameter (100 nm) surface and over segments of 3 nm in the case of the small diameter (15 nm) surface, whereas, on the corresponding 100 nm and 15 nm void regions, the binding coefficient was zero. The binding coefficient was nonzero and uniformly distributed throughout the flat surface. The direct interaction term was nonzero only on the flat and the 15 nm nanotubular surface. The values of the diffusion constant, and bending modulus were selected according to experimental data (15,16). The restoring cytoskeleton spring constant φ_s_ was chosen arbitrarily to prevent vertical drift of the membrane segment during the simulation. The ω and direct interaction Bragg-Williams term (Ω) were chosen in the range that facilitates dynamic instability (ie, the growth of membrane protrusions). The range of stability and dynamic instability for these parameters was explored as described in previous theoretical studies (13,14,17).

## Results

Under anodization conditions used in this study the internal TiO_2_ tube diameter was 50 nm and 100 nm, while the length of the TiO_2_ nanotubes was up to 10 µm (Figure 1a)

**Figure 4:**
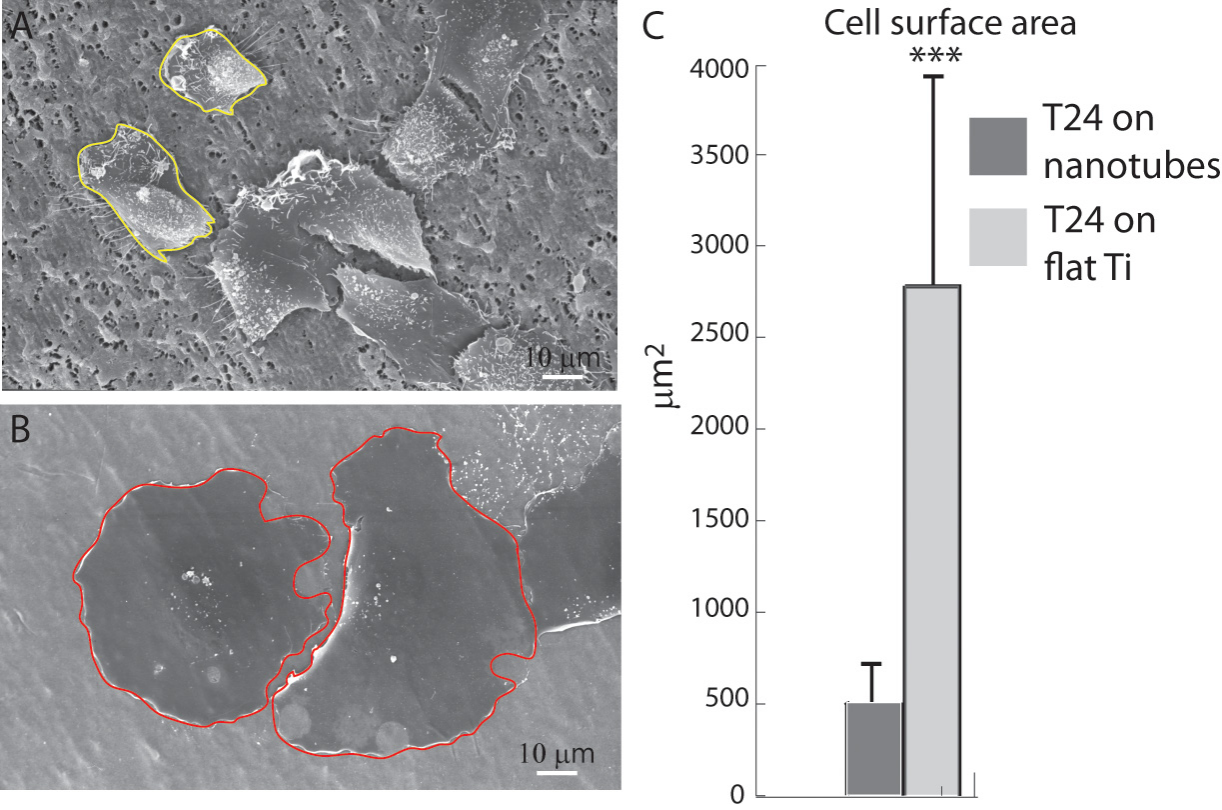
Summary statistics of changes in surface area of T24 cells grown on large diameter (50-100 nm) nanotubular TiO_2_ surfaces and on flat titanium surfaces. Note the clear difference in size between the traced cells (examples in yellow) on the nanotubular surface (**A**) in comparison to the cells (examples in red) on the flat titanium surface (**B**). Statistical analysis showed a significant (*P* < 0.0001***) increase in the surface area of T24 cells on flat surface when compared to T24 on 50-100 nm nanotubes (**C**). Data are reported as mean ± standard deviation.

The morphological alterations following the growth of cells on the large diameter TiO_2_ nanotubular and flat Ti surfaces were imaged using scanning electron microscope (Figures 2-3). TiO_2_ nanotubular surface cells exhibited a small cell diameter and numerous membrane protrusions, whereas the cells grown on the flat Ti surface had a large diameter and few membrane protrusions (Figure 4). The same experimental procedure was repeated on a surface that was one half Ti flat and one half constructed into TiO_2_ nanotubes (Figure 3). The boundary between these two regions also marked the boundary for the above mentioned morphological alterations. The cells growing on the flat part of the surface were larger in size and had fewer membrane exvaginations than the cells growing on the nanotubular part of the surface.

To evaluate the differences in the geometry, the surface area of the cells in the two cell groups were measured using ImageJ. The number of analyzed cells was 54 from the nanotubular group and 15 from the flat titanium group. Roughly the same number of micrographs were taken for both types of samples at the same magnification. Thus, there were more smaller cells on the same number of figures in comparison to larger cells. Summary statistics for the differences in the surface area is shown in Figure 4. The difference in the surface area was highly significant (*P* < 0.0001). The average surface area of T24 cells on nanotubes was only 487 μm^2^, whereas the average surface area of T24 cells on the flat titanium surface was 2744 μm^2^ (standard deviations, 191 μm and 1303 μm, respectively).

The vertical intersection of TiO_2_ nanotubular surfaces was modeled explicitly as rectangular edge-like profiles. The widths of the repeating nanotube edges were 3 nm and 20 nm for the small and large diameter nanotubular surfaces, respectively. According to the diameter of the nanotubes, the corresponding void regions between the edges were 15 nm and 100 nm. Since the adhesion of integrins could occur at the nanotube edges, the binding (adhesion) coefficient was nonzero only at the membrane regions directly above the edges. On the other hand, in the case of the flat surface, the binding coefficient was nonzero throughout the cell membrane. The initial condition of the following numerical simulations is a small random perturbation in the integrin density. The membrane regions that grew and reached titanium surface were trapped to the end of the simulation. This membrane trapping could be due to strong electrostatic interactions of the nanotube surface with the cell membrane and due to the binding of membrane integrins to fibronectin molecules attached at the nanotube edges (7).

**Figure 5:**
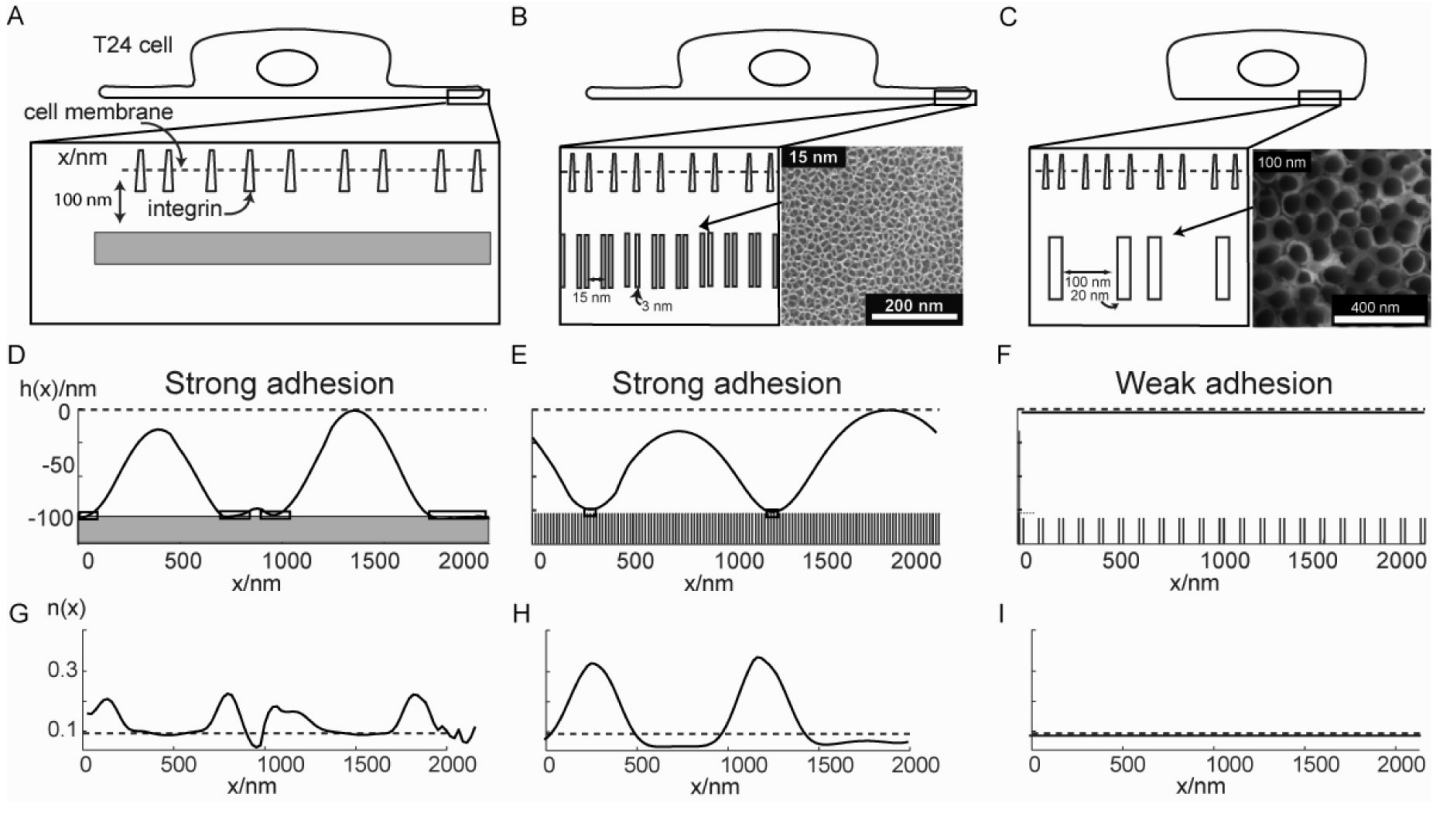
The effects of small diameter (15 nm) and large diameter (100 nm) nanotubular TiO_2_ surfaces, as well as flat (amorphous) titanium surfaces, on cell membrane adhesion. The modeled membrane segment distributed with integrins of positive spontaneous curvature is initially positioned 100 nm above the flat surface (**A**), the 15 nm diameter nanotube surface (**B**); see right inset), and the 100 nm diameter nanotube surface (**C**); see right inset). The membrane adhesion is driven by the negative adhesion energy and the positive spontaneous curvature of integrins. The membrane shapes and integrin density distributions are obtained by the minimization of the membrane free energy. Steady state membrane shapes h(x) (**D-F**) and integrin density distributions n(x) (**G-I**) of the cell membrane adhered to the corresponding surfaces are plotted. Note the cell membrane adhesion to the flat titanium surface and to the 15 nm TiO_2_ nanotube surface, in comparison to the hindered adhesion to the 100 nm TiO_2_ nanotube surface.

Numerical simulations demonstrated that the cell membrane adhered strongly to small diameter and flat surfaces, whereas a weak adhesion was observed to the large diameter surface (Figure 3). The steady state shapes reached a steady state in the time scale of minutes. The cell membrane adhered more to the flat surface and small diameter TiO_2_ nanotube surface than to the large diameter surface (see rectangular boxes in Figure 5A-C). The attraction of integrins toward positive membrane curvatures was responsible for a positive feedback loop, facilitating the membrane protrusive growth toward the underlying surface. In addition, the dynamic instability that was responsible for the membrane growth was driven by the negative binding energy of the integrins to the titanium surface. The density of integrins was highest at the regions making contact with the titanium surface.

## Discussion

The growth of T24 cells on large diameter (50-100 nm) nanotubular TiO_2_ surfaces revealed a significant reduction in the cell contact area as well as the enhanced growth of membrane exvaginations, in comparison to T24 cells grown on flat titanium surface. A simple computational model was constructed to explain the difference in the cell contact area. The model assumption was that low density of TiO_2_ nanotube edges on the large diameter TiO_2_ nanotubular surface presented less adhesion area than the one on the flat surface. The model investigated cell membrane adhesion to TiO_2_ surfaces of small diameter (15 nm) TiO_2_ nanotubes, large diameter (100 nm) TiO_2_ nanotubes, and a flat Ti surface. The membrane constituent was an integrin molecule, which could adhere and induce a positive membrane curvature. Numerical simulations demonstrated dynamic instability-driven adhesion mostly for the small diameter TiO_2_ nanotube and flat titanium surfaces, whereas the adhesion to the large diameter TiO_2_ nanotube surface was not facilitated (Figure 5). The integrin density was highest at the adhesion contacts of concave geometry and lowest at the indented membrane regions in between. To conclude, the smaller contact area of adhesion of the cell membrane to the large diameter TiO_2_ nanotube surface could be explained by the smaller density of TiO_2_ nanotube edges, which does not facilitate the adhesion-driven membrane growth. In the case of small diameter TiO_2_ nanotube surface, the voids are completely compensated by the increased strength of adhesion on TiO_2_ nanotube edges in comparison to the flat Ti surface (10).

The present experimental results illustrate morphological changes of human urothelial T24 cells on different TiO_2_ nanotubes compared to Ti scaffolds. Imaging of cell morphology with SEM revealed nanotubular intercellular membrane connections on large diameter nanotubular surface, in contrast to a Ti flat surface where intercellular membrane nanotubes were nearly completely absent. Similar intercellular membrane nanotubular structures were found previously also in weakly connected cells, such as immune cells and astrocytes, keratinocytes after long term TiO_2_ or ZnO treatment, or in cells that actively migrate, for example, cancer cells (18,19). It is possible that the weak adhesion of T24 cells to the large diameter TiO_2_ nanotubular surface might be accompanied by smaller contact adhesion area, and more spherical shape of the cells (ie, less flattened). Furthermore, the weak adhesion to the surface could be compensated by the growth of long membrane protrusions and intercellular membrane connections that increase the cell anchoring to the neighboring cells.

**Figure 6:**
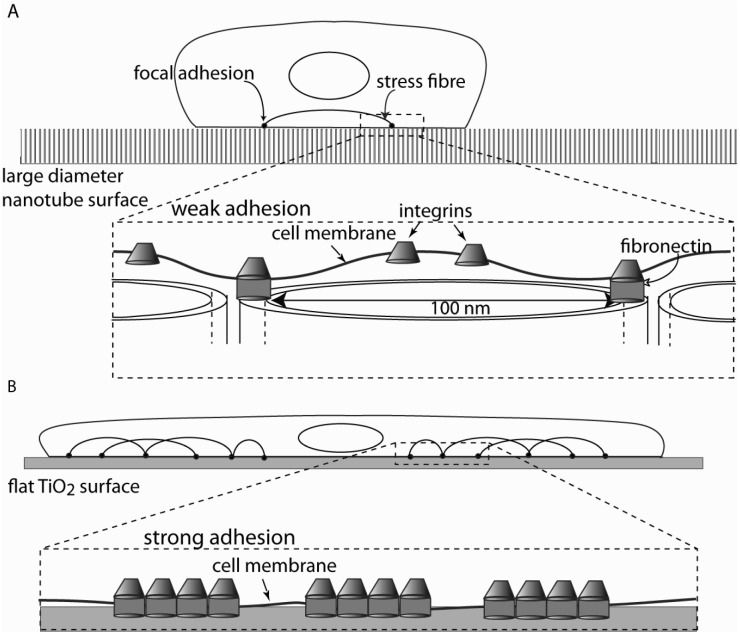
A possible mechanism underlying the weak adhesion of a T24 cell membrane to large diameter (100 nm) TiO_2_ nanotubular surfaces. The low density of TiO_2_ nanotube edges in the case of large diameter TiO_2_ surface presents less adhesion areas for integrins and fibronectins, and thus, the adhesion to 100 nm nanotubes is weak (**A**). Furthermore, the long distance (100 nm) between neighboring nanotubes may not facilitate the cross-interaction between integrins, which may be crucial for the formation of a focal adhesion (in close-up). On the flat titanium surface, the even distribution of integrins that make contact with the substrate surface may facilitate the formation of focal adhesions, and thus, explaining the observed increase in cell diameter and cell flattening (in close-up) (**B**).

Previous studies analyzed the effects of convex and concave surface edges of nanostructured TiO_2_ surface on the electric surface charge density and electrical field (7,10). Mathematically, convex edges of finite curvature would aggregate electrical charges and provoke larger electrical field, which is decreased with increasing distance from the edge (7). In contrast, the surface charge density and the electric field strength were smallest at the concave edges (7). Consequently, we proposed that the sharp edges of TiO_2_ nanotube surface aggregate electrical charges, which facilitate the strong binding of T24 cells to the sharp convex edges of small TiO_2_ diameter nanotubes (7-9). On the other hand, the area density of sharp edges of TiO_2_ nanotubes walls is smaller in the case of large diameter TiO_2_ nanotube surface, and thus does not facilitate the formation of focal adhesions and the cell flattening to the same extent as is the case with small diameter TiO_2_ nanotube surface (Figure 6).

To conclude, the observed small cell contact area on the large diameter (50-100 nm) TiO_2_ nanotube surface could be due to the weak adhesion to the TiO_2_ nanotube surface, whereas, the strong cell adhesion to the flat titanium surface might drive the cell flattening. More detailed studies of the growth of benign RT4 cancer cells should be performed on different titanium nanostructured scaffolds. Using fluorescent labels for actin filaments and integrins, the predicted changes in the adhesion strength can be investigated. The aggregation of negative electrical charges at highly curved edges (7), such as the nanotubular edges of TiO_2_ nanotube surface, should be analyzed using positively charged fluorescent dyes. We hope that the study of shape transformation of malignant cancer cells on different nanostructured surface may set the ground for the development of the novel intervention strategies in cancer treatment (20,21).

## References

[1] Engler AJ, Sen S, Sweeney HL, Discher DE (2006). Matrix elasticity directs stem cell lineage specification.. Cell.

[2] Yim EK, Reano RM, Pang SW, Yee AF, Chen CS, Leong KW (2005). Nanopattern-induced changes in morphology and motility of smooth muscle cells.. Biomaterials.

[3] Ponche A, Bigerelle M, Anselme K (2010). Relative influence of surface topography and surface chemistry on cell response to bone implant materials. Part 1: physico-chemical effects.. Proc Inst Mech Eng H.

[4] Anselme K, Ponche A, Bigerelle M (2010). Relative influence of surface topography and surface chemistry on cell response to bone implant materials. Part 2: biological aspects.. Proc Inst Mech Eng H.

[5] Stevens MM, George JH (2005). Exploring and Engineering the Cell Surface Interface.. Science.

[6] Matschegewski C, Staehlke S, Loeffer R, Lange R, Chai F, Kern DP (2010). Cell architecture-cell function dependencies on titanium arrays with regular geometry.. Biomaterials.

[7] Gongadze E, Kabaso D, Bauer S, Slivnik T, Schmuki P, van Rienen U (2011). Adhesion of osteoblasts to a nanorough titanium implant surface.. Int J Nanomedicine..

[8] Park J, Bauer S, von der Mark K, Schmuki P (2007). Nanosize and vitality: TiO2 nanotube diameter directs cell fate.. Nano Lett.

[9] Park J, Bauer S, Schmuki P, von der Mark K (2009). Narrow window in nanoscale dependent activation of endothelial cell growth and differentiation on TiO2 nanotube surfaces.. Nano Lett.

[10] Gongadze E Kabaso D Bauer S Park J Schmuki P Iglic A. Adhesion of osteoblast to a vertically aligned TiO2 nanotube surface - a mini review Mini Rev Med Chem 2012Aug 27. [Epub ahead of print] 22931535

[11] Visnjar T, Kocbek P, Kreft ME (2012). Hyperplasia as a mechanism for rapid resealing urothelial injuries and maintaining high transepithelial resistance.. Histochem Cell Biol.

[12] Kabaso D, Shlomovitz R, Auth T, Lew VL, Gov NS (2010). Curling and local shape changes of red blood cell membranes driven by cytoskeletal reorganization.. Biophys J.

[13] Kabaso D, Gongadze E, Perutkova S, Kralj-Iglic V, Matschegewski C, Beck U (2011). Mechanics and electrostatics of the interactions between osteoblasts and titanium surface.. Comput Methods Biomech Biomed Engin.

[14] Kabaso D, Shlomovitz R, Schloen K, Stradal T, Gov NS (2011). Theoretical model for cellular shapes driven by protrusive and adhesive forces.. PLOS Comput Biol.

[15] Grati M, Schneider ME, Lipkow K, Strehler EE, Wenthold RJ, Kachar B (2006). Rapid turnover of stereocilia membrane proteins: evidence from the trafficking and mobility of plasma membrane Ca2‏-ATPase 2.. J Neurosci.

[16] Evans E, Heinrich V, Ludwig F, Rawiczy W (2003). Dynamic tension spectroscopy and strength of biomembranes.. Biophys J.

[17] Kabaso D, Bobrovska N, Gozdz W, Gov N, Kralj-Iglic V, Veranic P (2012). On the role of membrane anisotropy and BAR proteins in the stability of tubular membrane structures.. J Biomech.

[18] Kocbek P, Teskac K, Kreft EM, Kristl J (2010). Toxicological aspects of long-term treatment of keratinocytes with ZnO and TiO2 nanoparticles.. Small.

[19] Frank M, Sodin-Semrl S, Rozman B, Potocnik M, Kralj-Iglic V (2009). Effects of low-molecular-weight heparin on adhesion and vesiculation of phospholipid membranes – a possible mechanism for the treatment of hyper coagulability in antiphospholipid syndrome.. Ann N Y Acad Sci.

[20] Kralj-Iglic V (2012). Stability of membranous nanostructures: a possible key mechanism in cancer progression.. Int J Nanomedicine.

[21] Sustar V, Bedina-Zavec A, Stukelj R, Frank M, Bobojevic G, Janša R (2011). Nanoparticles isolated from blood–a reflection of vesiculability of blood cells during the isolation process.. Int J Nanomedicine.

